# Exposure to Prenatal Stress Is Associated With an Excitatory/Inhibitory Imbalance in Rat Prefrontal Cortex and Amygdala and an Increased Risk for Emotional Dysregulation

**DOI:** 10.3389/fcell.2021.653384

**Published:** 2021-06-01

**Authors:** Francesca Marchisella, Kerstin Camile Creutzberg, Veronica Begni, Alice Sanson, Luis Eduardo Wearick-Silva, Saulo Gantes Tractenberg, Rodrigo Orso, Érika Kestering-Ferreira, Rodrigo Grassi-Oliveira, Marco Andrea Riva

**Affiliations:** ^1^Laboratory of Psychopharmacology and Molecular Psychiatry, Department of Pharmacological and Biomolecular Sciences, University of Milan, Milan, Italy; ^2^Developmental Cognitive Neuroscience Lab, Brain Institute, Pontifical Catholic University of Rio Grande do Sul, Porto Alegre, Brazil; ^3^Translational Neuropsychiatry Unit, Department of Clinical Medicine, Aarhus University, Aarhus, Denmark; ^4^Biological Psychiatry Unit, IRCCS Istituto Centro San Giovanni di Dio Fatebenefratelli Brescia, Brescia, Italy

**Keywords:** prenatal stress, anhedonia, anxiety, neuroscience, stress, resilience

## Abstract

Epidemiological studies have shown that environmental insults and maternal stress during pregnancy increase the risk of several psychiatric disorders in the offspring. Converging lines of evidence from humans, as well as from rodent models, suggest that prenatal stress (PNS) interferes with fetal development, ultimately determining changes in brain maturation and function that may lead to the onset of neuropsychiatric disorders. From a molecular standpoint, transcriptional alterations are thought to play a major role in this context and may contribute to the behavioral phenotype by shifting the expression of genes related to excitatory and inhibitory (E/I) transmission balance. Nevertheless, the exact neurophysiological mechanisms underlying the enhanced vulnerability to psychopathology following PNS exposure are not well understood. In the present study, we used a model of maternal stress in rats to investigate the distal effects of PNS on the expression of genes related to glutamatergic and GABAergic neurotransmissions. We inspected two critical brain regions involved in emotion regulation, namely, the prefrontal cortex (PFC) and the amygdala (AMY), which we show to relate with the mild behavioral effects detected in adult rat offspring. We observed that PNS exposure promotes E/I imbalance in the PFC of adult males only, by dysregulating the expression of glutamatergic-related genes. Moreover, such an effect is accompanied by increased expression of the activity-dependent synaptic modulator gene *Npas4* specifically in the PFC parvalbumin (PV)-positive interneurons, suggesting an altered regulation of synapse formation promoting higher PV-dependent inhibitory transmission and increased overall circuit inhibition in the PFC of males. In the AMY, PNS more evidently affects the transcription of GABAergic-related genes, shifting the balance toward inhibition. Collectively, our findings suggest that the E/I dysregulation of the PFC-to-AMY transmission may be a long-term signature of PNS and may contribute to increase the risk for mood disorder upon further stress.

## Introduction

Pregnancy is a crucial period in the life of mammals, and awareness is rising among the public for the potentially dangerous outcomes of exposure to stress during pregnancy on the developing fetal brain (Bleker et al., 2019). Maternal stress can encompass a variety of stressors, ranging from traumatic life events, natural disasters, or symptoms of anxiety and depression. Evidence from animal studies suggests that stress in the last week of pregnancy leads to alterations in offspring’s neurodevelopmental trajectories, with adverse cognitive and behavioral consequences, later in life (Hodes and Epperson, 2019). However, sex-specific neurobehavioral outcomes have been reported for rodents and humans (for reviews, see Weinstock, 2007; Bale, 2016). Nonetheless, not all offspring are affected by prenatal stress (PNS), and intrinsic factors such as genetic or molecular factors can also play a critical role in determining the functional outcomes (Abbott et al., 2018). More in detail, long-lasting changes in brain regions, such as the amygdala (AMY), prefrontal cortex (PFC), and hippocampus can occur during key gestational periods and are reported in humans (Hay et al., 2019). These regions, especially the PFC and AMY, are strongly interconnected; and their work in concert is essential to regulate mood, emotions, and stress responsiveness (Murray et al., 2011; Likhtik et al., 2014). Also, animal studies suggest that the “rapidly evolving” PFC of infants is highly susceptible to early adverse experiences and may be permanently shaped by early environmental factors, ultimately affecting function and subjects’ behaviors (Hodel, 2018). In particular, in rodents, exposure to PNS has been shown to modify dendritic morphology and synaptic connectivity of the PFC (Mychasiuk et al., 2012). A sexual dimorphic effect of perinatal stress in the PFC has also been observed for rats, with males being more prominently affected in spine density and anxiety-related behaviors (De Melo et al., 2018).

As for the AMY, a significant association between maternal depression in pregnancy and axonal organization in this region, measured with fractional anisotropy and axial diffusivity, has been reported in human male and female subjects (Rifkin-Graboi et al., 2013), whereas maternal cortisol concentrations during pregnancy were significantly associated with stronger neonatal AMY connectivity in girls and weaker connectivity in boys (Graham et al., 2019), as well as increased behavioral reactivity in female infants and less reactivity in male infants (Braithwaite et al., 2017). Moreover, sexually dimorphic outcomes of PNS and AMY structures have been described: higher maternal cortisol levels in earlier gestation were linked to a larger AMY volume in girls and not in boys, along with more affective problems in girls, partly mediated by AMY volume (Buss et al., 2012), and are thought to be related to the high density of gonadal steroid and glucocorticoid receptors (GRs) in this brain region, which may drive such sex differences in structural changes (Gray and Bingaman, 1996). Adult male rats that were exposed to PNS exhibited different developmental trajectories of the amygdalar nuclei (Kraszpulski et al., 2006) and display a larger AMY along with glial and neuronal cells in the lateral portion (Salm et al., 2004). Changes in PFC and AMY connectivity have been linked to stress resilience and emotional regulation (Wager et al., 2008). The PFC sends projections to both pyramidal and inhibitory neurons within the AMY (Cho et al., 2013; Hübner et al., 2014). Stimulation of the PFC is more potent at activating the basolateral interneurons leading to a short latency inhibitory postsynaptic potential in the pyramidal neurons (Rosenkranz et al., 2003). Thus, the PFC is thought to be a top-down negative modulator of the AMY activity, a circuitry that has been shown to be highly implicated in stress responsiveness and emotion regulation (Motzkin et al., 2015; Dulka et al., 2020).

A fine balance between excitatory and inhibitory (E/I) neurotransmissions is crucial for correct information receiving, processing, integration, and responsiveness by the PFC (McKlveen et al., 2019). Cortical E/I imbalance has been associated with stress-related disorders (Luscher et al., 2011), and PNS-derived E/I defects seem to involve impaired neuronal migration during fetal neurodevelopment, where precise control of the migration and placing of both E/I neurons is vital for the formation of balanced circuits in the brain (Hwang et al., 2019). Nonetheless, recent findings emphasize the prominence of GABA in initiating the effect of PNS in E/I balance (Fine et al., 2014) and shaping prefrontal–AMY connectivity, with higher GABA content in the medial PFC resulting in lower inhibition in the AMY (Delli Pizzi et al., 2017).

With these premises, here, we aimed at elucidating the long-lasting molecular effects of PNS exposure related to anxiety- and depressive-like behaviors of adult male and female rats. Since we hypothesized that the programming effects of PNS take time to fully emerge, we focused on adult behavior. We subjected pregnant Wistar rats to a restraint stress protocol from gestational day 14 to 21, and then we assessed male and female offspring behavior at postnatal day (PND) 80, as we expected alterations of anxiety- and/or depressive-like traits as well as sociability. We examined E/I dynamics through the investigation of transcriptional markers in both the PFC and AMY, complementing gene expression, and histological analyses.

## Materials and Methods

### Animals and the Restraint Stress Protocol

**FIGURE 1:**
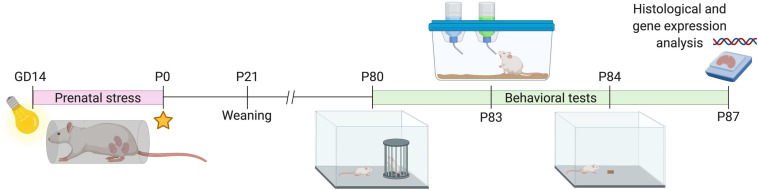
Schematic drawing of the experimental timeline. GD, gestational day; P, postnatal day. Cartoons for behavioral tests represent the following: P80, social investigation test; P83, sucrose preference; P84, novelty-suppressed feeding test.

A timeline for the experimental procedure is shown in Figure 1 (created with BioRender.com). Twenty nulliparous female Wistar rats were mated overnight with conspecific males from the same strain. Pregnant rats were housed in groups of two until day 14 of gestation when they were housed individually. Pregnant females were then randomly assigned to gestational stress or control group (*n* = 10 in each group). Restraint stress was applied from gestational day 14 to day 21, as previously described (Luoni et al., 2014, 2016; Panetta et al., 2017). Briefly, three stress sessions were performed each day (45 min each session, starting at 09:00, 12:00, and 17:00 ± 2 h) and during which animals were placed into transparent plastic cylinders (length = 20 cm; diameter = 9 cm; height = 9 cm) and exposed to bright light (1,500 lux). Dams were weighted on alternative days during the stress exposure to monitor stress responsiveness, and data are shown in Supplementary Figure 1b. At the end of the procedure, two out of the 10 stressed dams turned out not to be pregnant, and two ate the pups. Therefore, a total of six stressed dams could be included in the study. For the control group of dams, seven were finally included in the group, as the remaining three were not pregnant. Details on final litter sizes and sex ratios can be found in Supplementary Figure 1. Control dams were left undisturbed in home cages. Pups were left undisturbed in home cages to prevent unnecessary manipulations and weighted at PND 1, 14, and 21 when they were also weaned and housed in a group of 2/3 per cage under a 12/12-h light/dark cycle (lights on at 06:00 h), with *ad libitum* access to food and water in a temperature and humidity-controlled room (21 ± 1°C, 55 ± 5%, respectively). Cage cleaning was performed once a week by the animal facility staff throughout the experimental period. The experimental behavioral assessment was carried out during adulthood, from PND 80 to 87, between 10:00 and 17:00 h. All animal maintenance and experimentation procedures were approved by the Ethics Committee on Animal Use (CEUA) of PUCRS, Brazil under protocol #8922. Experiments were conducted in strict accordance with the National Institutes of Health Guide for the Care and Use of Laboratory Animals (NIH Publications No. 8023, revised 1978) and the International Council for Laboratory Animal Science (ICLAS).

### Novelty-Suppressed Feeding Test

The paradigm assesses anxiety-like behaviors in rodents by measuring the latency of an animal to approach and eat a familiar food in an aversive environment (Shephard and Broadhurst, 1982). Following ∼12-h food deprivation, at PND 84, rats were released in the corner of a brightly illuminated testing arena (100 cm × 100 cm), where a single pellet of food was previously weighted and located in the center. The time to approach and eat the pellet was measured within a 5-min period; however, time was stopped whenever the animal bites the food. Animals that did not bite the pellet within the 5-min session were not included in the data. Data analysis was performed by using two-way ANOVA, evaluating effect of PNS, effect of sex and interaction on raw measures, without any data transformation.

### Social Investigation Test

The social investigation test was used to assess approach-avoidant behavior as a measure of anxiety-like trait (File and Seth, 2003). Male and female rats (PND 80) born to either control or stress group of dams were tested for spontaneous social investigation. We used an adapted version of the protocol reported by Orso et al. (2018). The test was conducted in an open field (OF) arena (100 cm × 100 cm × 18 cm), and each session lasted for 7 min. During the first 3 min (habituation phase), rats were placed inside the arena and allowed to freely explore the novel environment, which included an empty, perforated black enclosure (25 cm length × 12 cm width × 8 cm height). Subsequently, rats were returned to home cage for 1 min, while an unfamiliar control rat of the same breed, age, and sex was placed inside the perforated enclosure, which allowed sensorial and nose contact between the animals but prevented them from touching each other. The test rat was returned to the testing arena (testing phase), and behavior was recorded for 3 min. The amount of time spent in the perimetral area immediately around the perforated enclosure exploring, touching, or sniffing the enclosure was manually measured and analyzed from recorded videos during both habituation and testing phases. The total time spent in the opposite corners to the enclosure during both habituation and testing phase was obtained, as well as the time spent physically interacting with nose-to-nose contact with the conspecific rat during the testing phase. Data were expressed as cumulative time in seconds, or percentage of time spent in the target area during the testing phase/time spent in the target area during the testing phase + time spent in the target area during the habituation phase. Data analysis was performed by using two-way ANOVA evaluating the effect of PNS, main effect of sex, and their interaction on raw measures, without any data transformation.

### Sucrose Preference Test

The sucrose preference test was used to assess anhedonia-like behavior and performed as previously (Guidotti et al., 2013; Calabrese et al., 2016). Beginning at PND 81, animals were singly housed and habituated to having two bottles of water in home cage. The night before the testing day (PND 83), all animals were water deprived for 12 h. On the day of testing, control and prenatally stressed rats were given the choice between a bottle containing normal drinking water and another containing a 1% sucrose solution. The bottles were left for 3 h and position swapped halfway through the test. Data were expressed as raw measures of sucrose intake in grams or transformed in sucrose preference calculated as a percentage of sucrose intake over the total fluid intake (water + sucrose). Data were analyzed using two-way ANOVA evaluating the main effect of PNS, main effect of sex, and their interaction.

### Maternal Behavior

Maternal care and behavior are known to crucially impact the offspring neurodevelopment. Stressed dams and relative control dams were assessed in their home cages every other day from PND 1 to PND 15. Maternal behavior was monitored every 2 min over a 20-min observation period at 09:00, 13:00, and 17:00 h. Behaviors were categorized as pup-directed (dams building the nest, in the nest nursing in any position, licking pups, contact with pups, retrieving the pups, or no interaction with pups) or self-directed (eating/drinking, self-grooming, off, or exit from the nest) (Champagne et al., 2003). The observations were quietly performed in the housing room, where the observer sat directly in front of the cages. Animals were not handled or disturbed whatsoever during this procedure. The index of maternal behavior was calculated by summing the total number of events for each specific behavior over the observation period.

### Open Field Test

Novelty-induced locomotor activity was recorded and further analyzed (ANY-maze). To explore the novelty factor, animals were not previously exposed to the arenas (100 cm × 100 cm), and the apparatuses were cleaned with 70% alcohol solution between trials to prevent transmission of olfactory cues. Spontaneous locomotor activity was monitored for 5 min, and the following parameters were scored: total distance moved, time spent in the center or periphery of the arena, and numbers of entries in the center and periphery of the arena (Schipper et al., 2011; Manfré et al., 2017). Center was defined as a 25 cm × 25 cm square right in the middle of the arena. Data analysis was performed by using two-way ANOVA evaluating the effect of PNS, main effect of sex and their interaction on raw measures, without any data transformation.

### Euthanasia and Brain Tissue Collection

All animals were euthanized by decapitation 2 days after the last behavioral test. The brains were quickly removed from the skull on an ice-chilled plate; and with the use of a scalpel, the brain was divided based on two halves of hemispheres.

Left hemispheres were immediately transferred to an ice-cold tube pre-filled with 4% paraformaldehyde (PFA) for fixation. Tubes were kept at 4°C, and after 24 h, PFA was changed to 30% sucrose for cryopreservation until the sinking of the brains to the bottom of the tube was observed (24-48 h). Subsequently, brains were transferred to a disposable Cryomold, embedded in optimal cutting temperature (OCT), and snap-frozen by submerging the block in 2-methyl butane cooled with dry ice for about 15 s. Samples were then removed, wrapped in aluminum foil, and stored at –80°C until sectioning.

Right hemispheres were dissected onto an ice-chilled plate. The PFC and AMY were isolated using a free-hand dissection approach with tweezers and scalpel according to Paxinos and Watson atlas coordinates (Paxinos and Watson, 2007). Following dissection, samples were instantly frozen in dry ice and stored at –80°C until molecular analysis.

### RNAscope *in situ* Hybridization Assay

Fixed-frozen brains were sectioned at cryostat (thickness: 10 μm). One out of six coronal sections were mounted on positively charged microscopic glass slides (Thermo Fisher Scientific). We used rat-peptidylprolyl isomerase B (Rn-Ppib, 313921) as a positive control probe to assess sample RNA quality; optimal permeabilization, a bacterial gene coding for dihydrodipicolinate reductase (Rn-DapB, 54684), as a negative control probe; and *Npas4* as target RNA probe (Rn-Npas4, 493881-C2). Probes were purchased from Advanced Cell Diagnostics (Hayward, CA, United States). All staining steps were performed following RNAscope protocols. Briefly, sections were defrosted and baked at 60°C for 30 min. Then, sections were permeabilized by antigen retrieval (5 min at 100°C) and incubated with protease mixture (30 min at 40°C). Probes were bound by incubation for 2 h at 40°C, chemically amplified, and then labeled by fluorophores [multiplex *in situ* hybridization (ISH)].

### Immunostaining

*Post hoc* immunofluorescence was performed immediately following ISH, incubating sections for 1 h at room temperature (RT) with bovine serum albumin (1%) in phosphate-buffered saline (PBS) containing Triton X-100 (0.4%). Slices were incubated for 72 h at 4°C with primary antibodies, as follows: 1:1,000 rabbit anti-parvalbumin (PV) (#NB120-1142, Novus Bio). Detection was obtained with Alexa dye-conjugated antibodies at a concentration of 1:500. Sections were cover-slipped with fluorescent mounting medium ProLong Gold Antifade reagent (Thermo Fisher Scientific) containing DAPI for nuclei visualization. Images were acquired with an LSM-900 confocal microscope (Carl Zeiss, Oberkochen, Germany) using a 10 × objective to navigate the area of interest and 60 × to snap the image. Images shown are typically from maximum projections of 6–8 × z-planes.

### Imaging and Quantification

The fluorescent signal was analyzed at 60 × magnification. Signal detection and unbiased quantification were performed using ImageJ (National Institute of Health) software. The experimenter was blind to the experimental group. PV-positive neurons were first identified as colocalizing with the DAPI and counted. RNA transcript signals of the gene probes (*Ppib* and *Npas4*) appeared as punctate dots in two distinct fluorescent channels (Chan et al., 2018). We considered nuclear staining to account for somatic localization of RNA transcripts/puncta signals and quantified the total number of puncta in every PV-positive cell, and the average number of puncta/cell was calculated (Erben et al., 2018). Occasionally, puncta can overlap and fuse appearing as clusters. Therefore, we quantified clusters by dividing the measured area of the cluster by the average area of five different puncta in the acquired field.

### RNA Extraction and mRNA Level Analysis

The extraction of total RNA from the PFC and AMY was performed as previously described (Marchisella et al., 2020). Briefly, samples were submerged into PureZol RNA isolation reagent (Bio-Rad Laboratories, Italy) and tissue disrupted in Qiagen TissueLyser II (Qiagen, West Sussex, United Kingdom). RNA concentration was measured at NanoDrop spectrophotometer and used for quantitative real-time polymerase chain reaction (qRT-PCR) (CFX384 real-time system, Bio-Rad Laboratories, Italy). Samples were run in triplicate, and β-actin was used as a reference. Standard deviation for considering triplicates reads technically reliable was set at 0.5. Examined genes and relative sequences are reported in Table 1. As many of the physiological and pharmacological properties of *N*-methyl-D-aspartate glutamate receptors (NMDARs) depend on the specific composition of the subunits (Cull-Candy and Leszkiewicz, 2004), we selected *Grin1*, *Grin2a*, and *Grin2b* genes to explore possible alterations due to PNS. In addition, *vGlut1* and *vGat* were analyzed, as in the mammalian nervous system the presence of either *vGlut1* or *vGat* defines E/I neurons, respectively, and their functioning is based upon the strict segregation of *vGlut1* or *vGat* expression (Edwards, 2007). We also estimated mRNA levels of the vesicle-associated GABA-synthesizing enzyme *Gad1/67* and *Arc/Arg 3.1*, *Zif-268*, and *Npas4*, as their neuronal gene expression is dynamically changed in response to neuronal activity (Gallo et al., 2018). Primers and probes were purchased from Eurofins MWG-Operon and Life Technologies. The raw values of threshold cycle (Ct) were first transformed into relative concentrations of the target gene relative to the endogenous control through the ΔΔCt method. Data were then expressed as fold change calculating the test samples relative to the control samples taking into account the efficiencies of amplification, according to Pfaffl (2001). For graphic clarity, data are expressed as a percentage of male control groups set at 100%. Data analysis was performed by using two-way ANOVA evaluating the effect of PNS, main effect of sex, and their interaction on data expressed as fold change.

**TABLE 1:** Sequences of forward and reverse primers and probes used in real-time PCR analyses.

Gene	Forward primer	Reverse primer	Probe
*Arc/Arg 3.1*	GGTGGGTGGCTCTGAAGAAT	ACTCCACCCAGTTCTTCACC	GATCCAGAACCACATGAATGGG
*Zif268*	GAGCGAACAACCCTACGAG	GTATAGGTGATGGGAGGCAAC	TCTGAATAACGAGAAGGCGCTGGTG
*Npas4*	TCATTGACCCTGCTGACCAT	AAGCACCAGTTTGTTGCCTG	TGATCGCCTTTTCCGTTGTC
*Vgat*	ACGACAAACCCAAGATCACG	GTAGACCCAGCACGAACATG	TTCCAGCCCGCTTCCCACG
*Vglut1*	ACTGCCTCACCTTGTCATG	GTAGCTTCCATCCCGAAACC	CTTTCGCACATTGGTCGTGGACATT
*Grin1*	TCATCTCTAGCCAGGTCTACG	CAGAGTAGATGGACATTCGGG	TGGGAGTGAAGTGGTCGTTGGG
*Gad1/67*	ACTTGGTGTGGCGTAGC	AGGAAAGCAGGTTCTTGGAG	AAAACTGGGCCTGAAGATCTGTGGT
β*-Actin*	CACTTTCTACAATGAGCTGCG	CTGGATGGCTACGTACATGG	TCTGGGTCATCTTTTCACGGTTGGC

### Composite Behavioral Score

We computed composite z-scores as an integrated overview of behavioral or molecular analysis. Behavioral z-score was calculated including only animals that actually ate during the novelty-suppressed feeding (NSF) test. Composite scores were obtained by averaging the z-scores of the latency to eat (in seconds) in the NSF, the sucrose preference, social investigation ratio, and six parameters of the OF test, namely, total distance traveled; number of entries in the periphery; time spent in the periphery; number of entries in the center; time spent in the center; and latency to enter the center (Guilloux et al., 2011; Peña et al., 2017; Cherix et al., 2020). The individual z-score per animal in each test was obtained by dividing the difference between each sample and the sample average by the sample’s standard deviation. The directionality of z-scores was adjusted so that decreased score values reflected lower emotional traits. Data analysis was performed by using two-way ANOVA (PNS × sex). This integrated analysis enables us to account for variation from the mean in subjects’ behavior across several tests, providing a more solid readout of individuals’ behavior in tests that we employed in this study and that we interpreted as sensitive to detect rat anxiety-like (Gould et al., 2009) and depressive-like (Willner et al., 1987) behaviors.

### Statistical Analysis

Data were analyzed and graphed in GraphPad Prism 8. The summary data for behavioral analysis are presented in the text as mean and SEM. For the gene expression set of data only, results are expressed as mean percent of control males not exposed to PNS and presented as mean ± SEM of independent determinations. Comparisons of datasets for behavioral and gene expression results were performed using two-way ANOVA, followed by Bonferroni’s *post hoc* test (as indicated), when appropriate. Survival analysis was used to analyze data from the NSF test and Mantel–Cox log rank test applied to evaluate differences between groups in this test. In addition, chi-square test was used to compare percentages of animals that ate or did not eat the chow of pellet during the NSF test. For RNAscope data, Student’s two-tailed *t*-test was used to compare the two conditions. Besides, to explore the relationship between behavioral performance in the NSF test and food consumption in the same test or alteration of gene expression in the AMY, Pearson correlation coefficient (r) analysis was performed including individual NSF test scores and corresponding quantity of food consumed or mRNA levels. The sample sizes used in this work are in agreement with those estimated by power analysis using G^∗^power software (power = 0.9, α = 0.05). For breeding, the sample size was originally of 20 rats. For behavior analysis on offspring, the original sample size was 17–26 rats. For NSF analysis and composite behavioral score, animals that did not consume the pellet were excluded from the two-way ANOVA, leading to a final sample size of 5–16 rats. For gene expression analysis, the original sample size was 17–26 rats. Exclusion criteria from the final analysis were due to either missing reads from qRT-PCR runs or standard deviation of three well triplicates higher than 0.5. For RNAscope experiments, the sample size was four to six rats. Differences were considered statistically significant at ^∗^*p* ≤ 0.05, ^∗∗^*p* < 0.01, ^∗∗∗^*p* < 0.001, and ^****^p < 0.0001. Non-significant trends were considered for *p*-Values between 0.06 and 0.08.

## Results

### Prenatally Stressed Adult Rats Show Subtle Differences in Emotional Behavior

**FIGURE 2:**
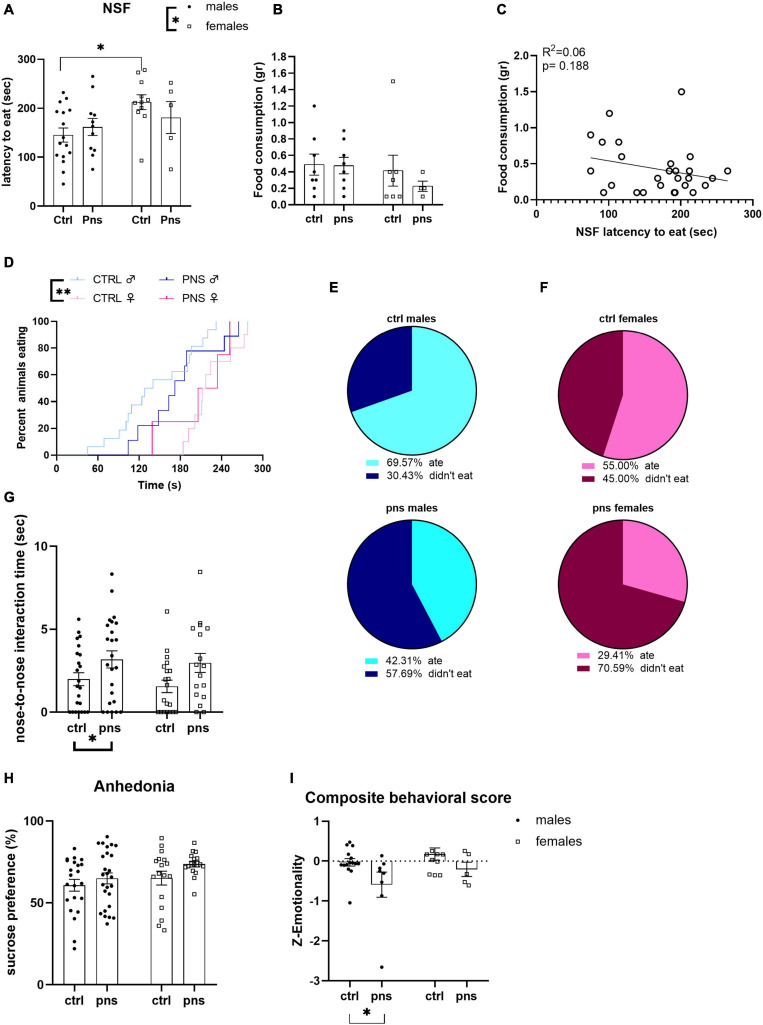
Analysis of anxiety- and depressive-like behaviors in prenatally stressed adult rats. (**A**) Latency to eat in a novel arena [novelty-suppressed feeding (NSF)] was affected by sex but not by prenatal stress (PNS). (**B**) Amount of food consumed during the 5-min test trial is shown. (**C**) Analysis by Pearson’s product–moment correlation (R^2^) between NSF score of latency to bite and food consumption during 5-min NSF test. Data are expressed as scatterplots, with each dot representing control or PNS male and female rats that ate the pellet during the test, and actual amount eaten could be quantified. The solid line represents the linear fit to all data points with a 95% confidence interval. (**D**) Survival plot of NSF latencies (Mantel–Cox log-rank test). (**E**) Pie charts indicate percentages (%) of male rats that ate or did not eat the pellet during the NSF test. Values in the upper chart represent eaters or not for control group. Lower chart shows percentage values for the PNS group (chi-square test). (**F**) Proportion of female rats that ate or did not eat the pellet during the NSF test. Values are the percentage (%) of eaters or not for control group in the upper chart and for the PNS group in the lower (chi-square test). (**G**) The effect of PNS on adult rats subjected to social investigation test was scored, and the total time of nose-to-nose interaction is shown. (**H**) The sucrose preference of control and PNS males and females is shown. (**I**) Anxiety- and depressive-like behavior computed as a composite z-score comprising latency to first bite in an unfamiliar environment, social investigation ratio, anhedonia, and distance traveled in the open field, as well as number of entries and time spent in the periphery and center of the open field. Data information: symbols represent individual data; error bars indicate mean ± SEM. For direct comparison of two groups: ^∗^*p* < 0.05; ^∗∗^*p* < 0.01 (two-way ANOVA with Bonferroni’s multiple-comparison test).

We assessed the behavioral phenotype by testing male and female rats exposed to PNS for anxiety-like behavior in the NSF test. Two-way ANOVA revealed no effect of stress [*F*(1, 39) = 0.1503, *p* = 0.7004], a significant effect of sex [*F*(1, 39) = 5.067, *p* = 0.0301], and no stress × sex interaction [*F*(1, 39) = 1.530, *p* = 0.2235]. Exploratory *post hoc* analysis showed that higher baseline anxious-like phenotype was observed for the females when compared with males (Figure 2A). There were no differences among groups in the total amount of food consumed during the test (Figure 2B), nor did we find any correlation between the latency to eat and the amount of pellet consumed (Figure 2C). Survival analysis indicated differences in latency to eat between control groups of male and female rats (log-rank Mantel–Cox: X ^2^ = 8.134, *p* = 0.004) (Figure 2D). As we noticed that many animals had to be censored from the analysis as they did not approach the pellet within the 300-s interval, we decided to approach the analysis by comparing the percentages of animals that ate the pellet and animals that took longer for each sex. Chi-square analysis trended toward an effect of PNS in reducing the percentage of male rats that consumed food during the NSF and increasing the percentage of animals that did not eat [*X*^2^ (1, *N* = 49) = 3.66, *p* = 0.05]. Pie charts are shown in Figure 2E. Contrarily, we did not observe differences when comparing percentages between the control and PNS groups of female rats [*X*^2^ (1, *N* = 37) = 2.45, *p* = 0.11] (Figure 2F).

Next, we assessed social behavior in the social investigation test, as an additional index of approach-avoidance behavior that is dependent on anxiety-like states in rodents (Lezak et al., 2017). We found a significant main effect of PNS when we scored the time the animals spent physically interacting (nose-to-nose) with the conspecific [*F*(1, 80) = 7.632, *p* = 0.0071] (Figure 2G), though no differences in the social investigation ratio, time spent in the interaction zone, and time spent in the corners opposite to the enclosure were found (Supplementary Figure 2).

We also investigated the anhedonic phenotype by scoring sucrose preference. The two-way ANOVA failed to show significant effects of PNS or sex, though there were trends for both [*F*(1, 78) = 3.29, *p* = 0.073; or *F*(1, 78) = 3.55, *p* = 0.063, respectively] (Figure 2H). Consistently, raw measures of liquid intakes showed a remarkable increase in sucrose consumption as opposed to water for all the animals tested (Supplementary Figure 3a), while, as expected, female rats displayed overall lower total fluid intake than males (Supplementary Figure 3b). Moreover, no effect of PNS was detected when we subjected our animals to OF test (Supplementary Figure 5).

Of note, we did not find any difference in any of the maternal behaviors between control rats and dams subjected to gestational restraint stress (Supplementary Figure 4a). We then grouped behaviors in pup-directed and self-directed behaviors; and analysis showed that both groups engaged in significantly more pup-directed behaviors than self-directed behaviors [effect of behavior type: *F*(1, 22) = 27.79, *p* < 0.0001] (Supplementary Figure 4b).

Lastly, we computed a behavioral composite score that combines data across interrelated tests, to gain a broader and more integrated estimate of how PNS impacts emotional behavior in adulthood. The composite score integrated data from NSF, sucrose preference, social investigation, and OF test. With two-way ANOVA, we found a main effect of stress [*F*(1, 35) = 6.059, *p* = 0.0189] toward lower emotional state, and no effect of sex nor interaction of the two factors (Figure 2J).

Altogether, our findings support the view that prenatal exposure to stress can mildly influence emotional-related behavior in adult offspring, at least at the baseline level.

### Sex Differences in the Expression Profile of Genes Related to Excitatory and Inhibitory Neurotransmissions in the Prefrontal Cortex

**FIGURE 3:**
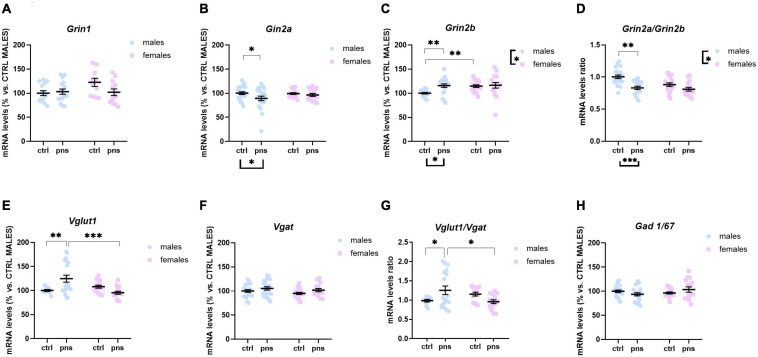
Analysis of (**A**) *Grin1*, (**B**) *Grin2a*, and (**C**) *Grin2b* mRNA levels; (**D**) *Grin2a:Grin2b* ratio; (**E**) *Vglut1* and (**F**) *Vgat* mRNA levels; (**G**) *Vglut1/vgat* ratio; and (**H**) *Gad 1/67* mRNA levels in the prefrontal cortex of male and female rats exposed to prenatal stress. Data are expressed as a percentage of control males not exposed to stress (set at 100%) and represent the mean ± SEM of at least 10 independent determinations. Data information: symbols represent individual data. ^∗^*p* < 0.05; ^∗∗^*p* < 0.01; ^∗∗∗^*p* < 0.001 vs. control males (two-way ANOVA with Bonferroni’s multiple-comparison test).

To explore the long-term influence of PNS on glutamate transmission in the PFC of male and female rat offspring, we measured the mRNA levels of *Grin1*, *Grin2a*, and *Grin2b* genes, as the functional properties of the NMDARs are strongly related to the type of subunit composition. Two-way ANOVA revealed no main effects of stress [*F*(1, 48) = 1.821, *p* = 0.1835] or sex [*F*(1, 48) = 2.686, *p* = 0.1078] on *Grin1* levels (Figure 3A), or stress × sex interaction [*F*(1, 48) = 3.397, *p* = 0.071], though there was a trend. *Grin2a* expression levels were decreased in PNS male rats, as compared with controls [main effect of stress, *F*(1, 76) = 3.859, *p* = 0.05; *post hoc* analysis *p* < 0.05] (Figure 3B), while no effect was found for the females. Conversely, we found a significant effect of stress [*F*(1, 74) = 5.859, *p* = 0.018] on Grin*2b* mRNA levels (Figure 3C), along with a significant effect of sex [*F*(1, 74) = 4.51, *p* = 0.037] and a significant stress × sex interaction [*F*(1, 74) = 3.789, *p* = 0.05]. Multiple comparison analysis showed significantly higher *Grin2b* mRNA levels for males exposed to PNS than for control males (*p* < 0.01). Furthermore, females show greater baseline expression of *Grin2b* than males (*p* < 0.01).

Key functional properties of the glutamate receptor can also be assessed by the relative expression of NMDAR subunits. In particular, an increase of *Grin2a* relative to *Grin2b* can be used as an index of the normal maturation that turns the NMDAR activity to faster kinetics (Myers et al., 2019). Thus, we quantified *Grin2a:Grin2b* ratio (Figure 3D), and we found a strong effect of stress [*F*(1, 68) = 19.72, *p* < 0.0001] and a significant effect of sex [*F*(1, 68) = 6.732, *p* = 0.011], but no significant interaction of the two factors [*F*(1, 68) = 3.313, *p* = 0.073], although a trend was detected. *Post hoc* analysis revealed that PNS exposure produced a significantly decreased ratio for males (*p* < 0.001), whereas no effect was observed for the females.

We also examined the expression levels of *Vglut1* and *Vgat* genes, the predominant glutamate and GABA vesicular transporters in the PFC, which may be considered a proxy for the integrity of glutamate and GABA neurons. For *Vglut1*, ANOVA revealed a trend effect of sex [*F*(1, 58) = 2.815, *p* = 0.0620] along with a strong interaction between stress and sex [*F*(1, 58) = 7.918, *p* = 0.0017]. *Post hoc* analysis showed increased expression for PNS males, relative to controls (*p* < 0.01) (Figure 3E), while no effect of PNS was detected in females. On the other hand, PNS did not alter *Vgat* expression levels in the PFC of neither sex, as shown in Figure 3F. We also quantified the *Vglut1*/*Vgat* ratio (Figure 3G), which reflects the E/I balance in the PFC (Fattorini et al., 2017), and we found a significant stress × sex interaction [*F*(1, 53) = 8.116, *p* = 0.0062]. *Post hoc* analysis revealed that *Vglut1*/*Vgat* ratio was significantly increased in PNS males (*p* = 0.05), while no differences were detected in the females.

Lastly, we assessed changes in the GABA-synthesizing enzyme *Gad 1/67*. Although two-way ANOVA showed a significant stress × sex interaction [*F*(1, 74) = 3.925, *p* = 0.05], no differences among groups were revealed by *post hoc* analysis (Figure 3H).

Taken together, at the molecular level, PNS appears to influence the baseline E/I balance of the PFC and to shift the NMDAR composition toward a more immature configuration, an effect that is prominent in males.

### Effects of Prenatal Stress on the Expression Levels of Activity-Dependent Genes in the Prefrontal Cortex

We next asked whether the observed effects of PNS on the E/I balance in the PFC would reflect altered expression profiles of activity-dependent immediate early genes (IEGs) in the same region. We measured the expression of *Arc/Arg 3.1*, *Zif-268*, and *Npas4*, three different markers of neuronal activation.

**FIGURE 4:**
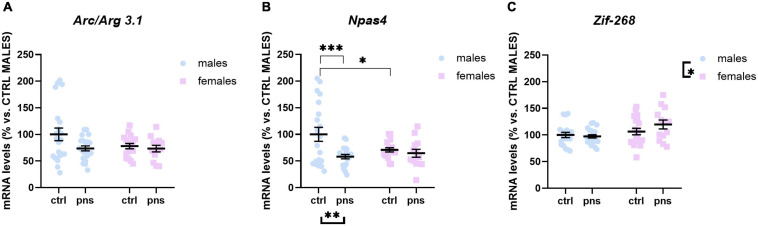
Analysis of (**A**) *Arc/Arg 3.1*, (**B**) *Npas4*, and (**C**) *Zif268* mRNA levels in the prefrontal cortex of males and females exposed to prenatal stress. Data are expressed as a percentage of control males not exposed to stress (set at 100%) and represent the mean ± SEM of at least 10 independent determinations. Data information: symbols represent individual data. ^∗^*p* < 0.05; ^∗∗^*p* < 0.01; ^∗∗∗^*p* < 0.001 vs. control males (two-way ANOVA with Bonferroni’s multiple-comparison test).

A*rc/Arg 3.1* expression was not significantly influenced by stress (main stress effect: F(1, 68) = 3.505, *p* = 0.0655), although a trend was detected (Figure 4A). *Npas4* mRNA levels were strongly regulated by PNS exposure [*F*(1, 66) = 7.612, *p* = 0.0075], with a significant stress × sex interaction [*F*(1, 66) = 4.115, *p* = 0.0465]. Multiple comparison analysis revealed decreased expression levels of *Npas4* gene in PNS males versus control (*p* < 0.01), and a lower baseline expression for females when compared with males (*p* < 0.05) (Figure 4B). Lastly, the expression of *Zif-268* was not affected by PNS exposure (Figure 4C), though a main effect of sex [*F*(1, 68) = 6.724, *p* = 0.0116] was found.

### Stress-Induced *Npas4* mRNA Upregulation Is Localized in Parvalbumin-Positive Interneurons of the Prefrontal Cortex

**FIGURE 5:**
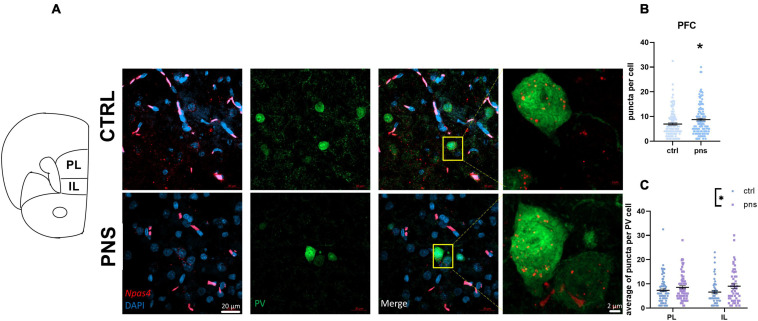
(**A**) Left: Schematic of adult rat bregma locations of prefrontal cortex subregions included in our analysis to selectively target the expression of *Npas4* in PV neurons. PL, prelimbic cortex; IL, infralimbic cortex. Right: Representative images from multiplexed RNA scope experiment aimed at detecting *Npas4* (red) expression in the parvalbumin-positive neurons (green) of the prefrontal cortex (PFC). Inset shows magnified portions depicted by the yellow box. (**B**) Quantification of *Npas4* expression across the PFC, *n* = 124–134 cells (data from 4–6 rats per group). Symbols represent individual data, *p < 0.05 (Student’s *t*-test). (**C**) Quantification of *Npas4* expression in PL, *n* = 72–81 cells, and IL, *n* = 49–52 cells (data from 4–6 rats per group). Symbols represent individual data, *p < 0.05 (two-way ANOVA).

Our data thus far support the view of a more prominent disturbance of E/I balance and activity in the PFC of PNS males. PV-positive interneurons are critical regulators of E/I balance in the PFC (Ferguson and Gao, 2018), and altered PV activity in this region has been reported to disinhibit excitatory transmission and underlie anxiety-like behavior (Berg et al., 2019). Thereby, to expand on whether the PNS-induced shift toward excitation stems from an effect of PNS on the PV cells, we performed immunohistochemical analysis to map *Npas4* expression within the PV neuronal population in naive and prenatally stressed males. Across the three activity-dependent transcription factors previously analyzed, *Npas4* is particularly relevant, as it is predominantly expressed in both E/I neurons of the PFC (Damborsky et al., 2015), and it regulates E/I balance by promoting synapse formation (Spiegel et al., 2014). We examined coronal sections of the PFC utilizing RNAscope ISH combined with immunofluorescence for specific PV cell labeling. Figure 5A shows representative photomicrographs of brain sections analyzed where *Npas4* mRNA was detected. As expected, puncta were observed in the nucleus (Figure 5A, insets). In contrast with our qRT-PCR finding, we found a significant upregulation of *Npas4* expression in PV cells in PNS rats with respect to controls (*t* = 2.538, *df* = 256, *p* < 0.05, Student’s *t*-test; Figure 5B). We further split *Npas4* puncta quantification into two subregions of the PFC: infralimbic (IL) and prelimbic (PL) cortices, to establish the specific contribution of these subregions, which have been associated with anxiety-like behavior (Suzuki et al., 2016; Berg et al., 2019). As shown in Figure 5C, two-way ANOVA revealed a significant effect of stress [*F*(1, 250) = 6.381, *p* < 0.05] apparently increasing *Npas4* expression in both IL and PL subregions; however, no significant differences were detected when PNS rats were compared with controls, indicating an equal effect of PNS on *Npas4* transcription in these areas.

Collectively, these data may suggest heightened region inhibition from PV cells of the PFC in male rats exposed to stress *in utero*, which may promote the observed shift of E/I balance.

### Stress-Induced Modifications of Markers of Excitatory and Inhibitory Neurotransmission in the Amygdala

**FIGURE 6:**
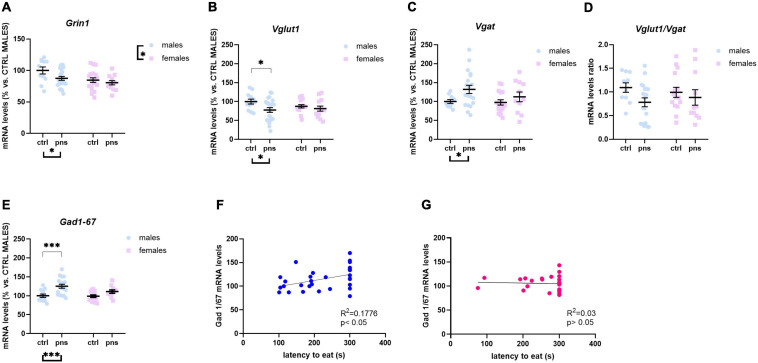
Analysis of (**A**) *Grin1*, (**B**) *Vglut1*, and (**C**) *Vgat* mRNA levels; (**D**) *Vglut1/vgat* ratio; and (**E**) *Gad 1/67* transcription levels in the amygdala of males and females exposed to prenatal stress. Data are expressed as a percentage of control males not exposed to stress (set at 100%) and represent the mean ± SEM of at least 10 independent determinations. Data information: symbols represent individual data. ^∗^*p* < 0.05; ^∗∗∗^*p* < 0.001 vs. control males (two-way ANOVA with Bonferroni’s multiple-comparison test). (**F**) Analysis by Pearson’s product–moment correlation (R^2^) between *Gad 1/67* messenger RNA levels and novelty-suppressed feeding (NSF) score of latency to bite. Data are expressed as scatterplots, with each dot representing either a control or PNS male rat for which *Gad 1/67* mRNA levels could be quantified. The solid line represents the linear fit to all data points with 95% confidence interval. (**G**) Analysis by Pearson’s product–moment correlation (R^2^) between *Gad 1/67* messenger RNA levels and NSF score of latency to bite. Data are expressed as scatterplots, with each dot representing either a control or PNS female rat for which *Gad 1/67* mRNA levels could be quantified. The solid line represents the linear fit to all data points with 95% confidence interval.

Next, we sought to ask whether transcriptional alterations detected in the PFC may also affect AMY E/I stability, as it receives sensory input primarily from the PFC, integrating and implementing signals before further transmitting to downstream regions (Wager et al., 2008). Two-way ANOVA on *Grin1* mRNA levels showed significant main effects of stress [*F*(1, 59) = 4.360, *p* = 0.0411] and sex [*F*(1, 59) = 7.739, *p* = 0.0072], but not stress × sex interaction [*F*(1, 59) = 1.068, *p* = 0.3055]. Exploratory *post hoc* analysis did not find significantly altered expression of the gene in PNS males vs. controls, although a trend was detected (*p* = 0.06) (Figure 6A). Additionally, while PNS exposure did not affect *Grin1* expression in females, the group exhibited lower baseline expression, than did control males (*p* < 0.05). We did not observe significant changes in *Grin2a* and *Grin2b* expression levels in either sex (data not shown). Moreover, in contrast with the changes observed in the PFC, the expression of *Vglut1* in the AMY was significantly decreased in PNS male rats, when compared with control animals [main effect of stress: *F*(1, 59) = 4.929, *p* = 0.0303; *post hoc* analysis (*p* < 0.05)] (Figure 6B), while no differences were observed between control and PNS females. With respect to *Vgat* expression, we found a main effect of stress [*F*(1, 52) = 5.122, *p* = 0.0278], with a trend toward an increase in male PNS rats (*post hoc* analysis, *p* = 0.06) (Figure 6C), while no difference was detected for the females. The *Vglut1/Vgat* ratio was not affected by PNS [main effect of stress: *F*(1, 51) = 3.120, *p* = 0.0833], although a trend was found (Figure 6D).

We also found a pronounced effect of stress on *Gad 1/67* mRNA levels [*F*(1, 52) = 15.94, *p* = 0.0002], with an increased expression in PNS males, as compared with their relative controls, while no difference was observed for the females (Figure 6E). Interestingly, correlation analyses revealed that latency to eat scores in the NSF test positively correlated with *Gad 1/67* expression levels in male rats (*r*^2^ = 0.1776, *p* < 0.05) (Figure 6F), but not in females (Figure 6G).

Altogether, these data suggest that PNS-induced disturbances of E/I dynamics in the AMY appear consistent in adult males, as compared with females.

### Basal Expression Levels of Activity-Dependent Genes in the Amygdala

**FIGURE 7:**
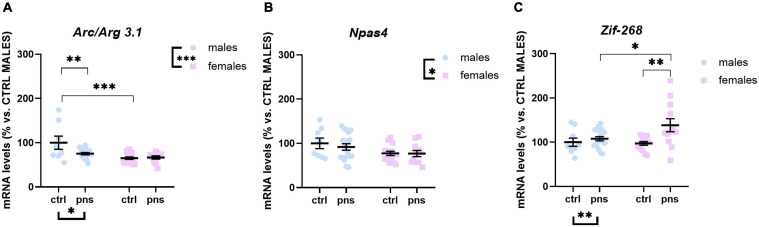
Analysis of (**A**) *Arc/Arg 3.1*, (**B**) *Npas4*, and (**C**) *Zif268* mRNA levels in the amygdala of males and females exposed to prenatal stress. Data are expressed as a percentage of control males not exposed to stress (set at 100%) and represent the mean ± SEM of at least 10 independent determinations. Data information: symbols represent individual data. ^∗^*p* < 0.05; ^∗∗^*p* < 0.01; ^∗∗∗^*p* < 0.001 vs. control males (two-way ANOVA with Bonferroni’s multiple-comparison test).

To examine whether such changes in E/I ratio within the AMY are associated with alterations of baseline activity, we analyzed the expression profile of *Arc/Arg 3.1*, *Npas4*, and *Zif268* as indicators of neuronal activation. In line with the changes observed within the PFC, two-way ANOVA revealed that PNS exposure downregulates *Arc/Arg 3.1* expression levels only in male rats [main effect of stress: *F*(1, 46) = 4.090, *p* = 0.0490; main effect of sex: *F*(1, 46) = 14.50, *p* = 0.0004; stress × sex interaction: *F*(1, 46) = 5.244, *p* = 0.0267; *post hoc* analysis *p* < 0.01] (Figure 7A). Moreover, baseline *Arc/Arg 3.1* mRNA levels were lower in females when compared with males (*p* < 0.001). Concerning the expression of *Npas4* mRNA levels in this region, we only observed an effect of sex [*F*(1, 48) = 5.894, *p* = 0.0190] (Figure 7B). Lastly, two-way ANOVA revealed a main effect of stress [*F*(1, 50) = 8.234, *p* = 0.0060] and a stress × sex interaction [*F*(1, 50) = 3.842, *p* = 0.05] for *Zif268* expression (Figure 7C), an effect that is driven by the higher mRNA levels in PNS females, as compared with control animals not exposed to PNS (*p* < 0.01).

## Discussion

In the present study, we sought to provide more evidence for the long-term effects of PNS to alter emotional behavior in adult male and female rats and to dysregulate E/I balance in the PFC and AMY, primarily through the modulation of glutamate-related genes. Importantly, we observed only a subtle effect of PNS on an integrated behavioral battery of anxious- and depressive-like traits. Our transcriptional analyses, however, point out to more obvious modifications in the PFC of males, via the altered excitatory drive and basal activation of the region. Furthermore, through the use of RNAscope ISH staining, we show that PNS exposure is associated with an upregulation of the *Npas4* gene in PV-positive neurons within males’ PFC. Lastly, gene expression analysis in the AMY of PNS animals shows an increased inhibitory drive in male rats, an effect that relates to high anxious-like latency to eat in the NSF test.

In the NSF test, we found clear differences in baseline behavior between control male and female rats, with no apparent effect of PNS. Control females took longer than males to feed in our setting. We, and others (Andrews et al., 2018; Prevot et al., 2019), operationally defined the latency to eat the pellet in the NSF test as an index of anxiety-like behavior. Our data are in line with basic sex differences described for exploratory behavior in a novel environment (Fernandes et al., 1999). However, conflicting findings on the relationship between the animals’ sex and this behavioral measure have also been reported (for review, see Kokras and Dalla, 2014). Strain variations and the impact of the estrous cycle in females on behavior as well as the testing conditions may also explain such basic differences in the literature. A limitation of our study is the lack of determination of estrous status in the female group, which may have influenced the behavior outcomes in the testing battery. It should be also pointed out that male and female rats have basic differences in energy metabolism (Leskanicova et al., 2020) and metabolomic profiling (Ruoppolo et al., 2018), with males showing increased energy metabolism, which could influence their decision-making process when faced with food stimulus. This metabolic standpoint is also reflected by differences in body weight that we detected starting from PND 43, with females exhibiting lower body weight, and overall lower drinking intake than males. While we cannot exclude that other parameters in the testing conditions could have impacted on the final data, for instance, longer food deprivation period, a smaller novel arena, or bright white food stage, we do not believe that the metabolic variables could have masked any possible effect of the PNS.

Moreover, changes in anxiety-like behaviors vary according to the specific threat encountered. Indeed, we did not detect differences among groups in the cumulative time spent in the center of the OF test, supporting the hypothesis that PNS animals only manifest a weak anxiety-like phenotype, at least at the basal level, which is when no other environmental challenges are introduced to evaluate their stress responsiveness. Indeed, there may be more behavioral phenotypes that have remained below the threshold of detection in our tests, and that might appear in other testing conditions, when PNS is combined with a further stressor such as a forced swim stress session or footshock. Interestingly, however, we detected a high number of animals that did not bite the pellet within the 5-min session of NSF; and when percentages of animals that ate and animals that did not were confronted, we observed an effect of PNS in males, increasing the number of non-eaters. Therefore, an additional interpretation of our NSF data could be that PNS animals simply selected a behavioral strategy that prioritized physical safety over their metabolic needs, which therefore may not reflect an increased anxiety-like trait at baseline but instead only a different strategy to reduce risk to their own life (Shansky, 2018). It is also important to point out that in this experiment, animals were tested during the light phase of the cycle, which may also represent a confounding factor in the interpretation of the data. Though it has been reported that stress impacts behavior also during the inactive period (Beeler et al., 2006; Morais et al., 2017), these animals should be tested during their active period as well, to rule out the discriminatory potential of the behavioral tests (Hossain et al., 2004). Moreover, we found an effect of PNS in the social investigation tests, when the time the animals spent physically interacting with the conspecific was scored. Although statistics failed to show clear differences, it appeared that stress increased nose-to-nose interaction only, but not any other proactive exploratory behavior of the interaction zone. Although the present study does not explore consequences of such aspect, on a purely speculative level, this may indicate altered social aggressive behavior, as only physical interaction was favored, but not any other cue of motivation to investigate the area that included the conspecific. The effect of PNS on social behavior is well studied in rodents (Patin et al., 2005; Lee et al., 2007), although just a few explore aggression (Kinsley and Svare, 1987; Ogrizek et al., 2018; Houwing et al., 2020). Nevertheless, De Souza et al. (2013) showed that prenatally stressed rats display more aggressive behavior measured as latency to first attack in the social interaction test.

We also observed a significant effect of PNS when a composite behavioral score was calculated, possibly decreasing the overall emotional state in prenatally exposed rats. Nevertheless, we did not find significant differences when the anhedonic state and maternal care were assessed. This could indicate that the subtle phenotypic traits observed in PNS offspring may be a consequence of accurate maternal care (Caldji et al., 1998), which could have partially reversed some priming effects of PNS.

Also, with respect to the translational value of this work, it is important to highlight that PNS emerges as a mere risk factor, not a determinant, of later mood disorder. We detect alterations at the molecular level in key regions implicated in emotional behavior that, however, are not sufficient to elicit behavioral alterations at baseline conditions. We cannot exclude of course the possibility that different choices for the behavioral battery could have facilitated the emergence of behavioral impairments. For instance, we chose to use a solution of 1% sucrose to assess anhedonia. However, the alternative of non-caloric saccharin could have also been an option. Nonetheless, by checking the raw measures of sucrose intakes, we observed that all animals drank more sucrose than water, thereby validating our experimental condition. In addition, choosing another behavioral readout for exploring the depressive-like dimension, such as the traditional forced swim test (FST), could have unmasked differences between control and PNS animals. While on the one hand this may be true, on the other hand, the FST could have masked the molecular alterations that we found at baseline, as it is known that exposure to a session of forced swim potently activates players and circuits that are otherwise silenced under resting conditions (Reed et al., 2012; Warden et al., 2012; Pitychoutis et al., 2014).

Nonetheless, identifying molecular differences in offspring brain structures associated with PNS exposure may allow the identification of subjects at risk for poor outcomes. Indeed, PNS animals (without an obvious phenotype) have been reported to respond differently to a stressful event later in life (second hit), supporting a cumulative model of stress effects on vulnerability to psychopathology (Vargas et al., 2016; Peña et al., 2017). PNS may set forth a cascade of effects in the developing brain including molecular, cellular, and network derangements, which may confer vulnerability to emotional disturbances that become manifest only after a further stress is encountered.

From a molecular standpoint, our study suggests that, particularly in male rats, PNS promotes E/I unbalance within the PFC and AMY, whose connectivity may play a role in stress vulnerability (Kumar et al., 2014).

In PNS males, we found an overall decreased *Grin2a*:*Grin2b* ratio in comparison with control animals. This is consistent with previous data in rats (Sun et al., 2013) and mice (Page and Coutellier, 2018) and suggests that PNS exposure may disturb the developmental switch of the GluN2B–GluN2A subunits in the PFC (Riva et al., 1997), leading to an immature network and synaptic phenotype that may play a role in the pathogenesis of the negative emotional state. Indeed, previous findings in humans show altered GluN2A and GluN2B expression levels in the PFC of depressed patients (Feyissa et al., 2009), while in rodents, GluN2B inhibition can prevent prefrontal hyperexcitability (Gupta et al., 2015). On the other hand, PNS did not produce major effects in the PFC of female offspring, possibly due to the different baseline expression of *Grin2b*, when compared with male rats. Such sex-dependent difference is consistent with previous work from our team (Fumagalli et al., 2009) and others (Kokras and Dalla, 2014; Weinstock, 2017). However, it should be also pointed out that the reproductive cycle of female rats has been shown to influence the GluN2A/GluN2B expression ratio (Gore et al., 2000), which may account for the absence of changes observed in our work. Together, this evidence suggests that the GluN2B subunit in males may be more sensitive to environmental stimuli, than in females, and that differences in GluN2B–GluN2A balance, arising developmentally during the GluN2B–GluN2A switch, may prompt some sex-related individual differences in stress response and risk for mood disorders.

Sex-related discrepancies were consistent for the vesicular transporters *Vglut1* and *Vgat* and their ratio (Wang et al., 2019) in the PFC, where PNS males showed increased levels of *Vglut1* expression and, consequently, higher *Vglut1*:*Vgat*, than did controls, which may foster dysregulation of the prefrontal glutamatergic system. On the other hand, females did not exhibit any effect of PNS on these genes in this region. Differences in expression levels of *Vglut1* upon PNS exposure are in line with previous data (Cao et al., 2018) and highlight sex-specific priming effects of *in utero* exposure to stress on the excitatory neurotransmission of the PFC. Collectively, data from our male rats seem to indicate that PNS is likely to elicit long-lasting effects onto glutamatergic signaling and its balance in the PFC. However, to gain more insights on whether such changes may be a result of alterations in the activity of PV-expressing fast-spiking interneurons within the PFC, our immunohistochemical analysis in males revealed a higher *Npas4* puncta signal in PV cells, suggesting increased excitatory synapse formation, which could promote a dysfunction of PV neurons. Indeed, it is well known that several neurodevelopmental disorders including mood disorders and schizophrenia feature abnormalities within the prefrontal GABAergic system (Luscher et al., 2011; Yizhar et al., 2011; Winkelmann et al., 2014). Thus, our data suggest that PNS may lead to higher PV-dependent inhibitory transmission and increased overall circuit inhibition in the PFC of males, similarly to the altered GABAergic transmission observed in stress-induced abnormal behaviors and psychiatric disorders in human patients (Maddock and Buonocore, 2012). This view also reconciles the lowered *Npas4* expression (and likely *Arc/Arg 3.1*, although only a trend) in the same group of rats and possibly accounts for a compensatory increase in *Vglut1* expression. These data are also consistent with studies showing prefrontal hypofunctionality in mice subjected to chronic mild stress (Shepard and Coutellier, 2018; Page et al., 2019). However, a number of studies have linked such hypofunctioning deficits to lower levels of PV interneurons in both rodents (Goodwill et al., 2018) and patients with psychiatric disorders (Hashimoto et al., 2003; Karolewicz et al., 2010). As PV expression is activity-dependent (Tropea et al., 2006), here, we used immunofluorescence to detect PV neurons with recent neuronal activity (*Npas4* expressing), which enabled their imaging and analysis. We also performed an estimation of the absolute number of PV-positive neurons in the PFC, and we found no differences between control and PNS groups (data not shown). This apparent contrast with the reported literature may be due to technical differences in the approach to estimate the total number or sensitivity of the antibodies used. Nevertheless, we believe that increased *Npas4* expression in GABAergic neurons of PNS rats fosters the formation of excitatory synapses onto these neurons to aid inhibition in an activity-dependent fashion (Spiegel et al., 2014). And we are confident that this finding does not depend on changes in the number of PV cells *per se*. It is thereby crucial to further dissect the exact impact of *Npas4* upregulation within the PV population at the cellular level using precise measures of PV cells neurophysiology but also at the circuit level to gain novel knowledge on the molecular mechanisms underlying prefrontal abnormalities in emotion regulation.

In both the PFC and AMY of PNS males, we observed the downregulation of *Arc/Arg 3.1* and *Npas4*, which we used to mark activity-dependent plasticity (Yap and Greenberg, 2018) and as downstream indicators of E/I dynamics upon exposure to PNS. A general decreased level of *Npas4* gene in the PFC is in line with reduced *Grin2a*:*Grin2b* ratio, indicative of lower plasticity, and may reflect defects of synaptic formation and function within neuronal populations other than the PV-expressing one. For instance, the pyramidal cells account for about 80% of all cortical neurons (Riga et al., 2014) and would be an interesting population to inspect in the future in this context. Conversely, no effect of PNS on the expression of these IEGs was detected for the females, which is remindful of the profound effect of sex on plasticity in the integrative cortico-amygdalar network.

We also sought to determine the extent to which prefrontal E/I imbalance could contribute to cortico-amygdalar dysfunctions and behavioral abnormalities observed in the PNS offspring. We postulated that stress-induced modifications in the functional activity of the PFC cells could result in AMY inactivation, as the PFC has been shown to directly inhibit the activity of AMY neurons (Quirk et al., 2003; Rosenkranz et al., 2003).

In the AMY of PNS males, we found lowered expression of *Vglut1*, along with a trend for *Grin1* and, on the other hand, increased mRNA levels of *Gad 1/67*. Remarkably, PNS males that did not approach the pellet during the NSF test promote a significant linear correlation with higher *Gad 1/67* transcription. The typical developmental regulation of emotional responses requires a transition from limbic–cortical system control over behavior (bottom-up) to cortico-limbic (top-down) (Casey et al., 2019). It has been shown that the impact of early life stress may promote an overactive state of limbic regions, such as the AMY, as well as a persistent “bottom-up” circuitry (Pattwell and Bath, 2017). However, this has also been considered an adaptive response to PNS, since the increased function of the AMY may reflect increased vigilance and more swift identification of danger (Lautarescu et al., 2020). Moreover, studies in humans have shown higher AMY reactivity in subjects exposed to early-life stress and presented with threatening images during the assessment (Swartz et al., 2015). Also, studies in rodents have reported the PNS effect on shifting a more excitatory configuration in the AMY of mice exposed to maternal separation (Ehrlich et al., 2015). Collectively, these data imply that increased GABAergic transmission in the AMY, in particular in the basolateral portion, is coupled to reduced anxiety-like behavior. In contrast, we find that high expression of *Gad 1/67* is associated with increased avoidance for food in the NSF test in PNS male rats. Consistently, a study using HAB mice, an animal model of increased trait anxiety, found markedly increased expression of the GABA synthesizing enzymes *Gad 1/65* and *Gad 1/67* in the AMY of these animals (Tasan et al., 2011). Such apparent discrepancy may be explained by a compensatory mechanism of increased GABA synthesis buffering the persistent derangement of limbic brain areas, including the altered E/I balance in the PFC. Interestingly, we did not observe prominent changes in transcriptional levels of E/I markers in PNS female AMY, nor correlations with behavior. Nevertheless, we cannot rule out that, although apparently intact, the AMY may be faulty in the female brain because of the contribution of other neurotransmitters, such as serotonin, acetylcholine, noradrenaline, or dopamine (Rosenkranz and Grace, 2001; Gillies et al., 2017; Huizink and de Rooij, 2018), as well as other brain regions for anxiety- and depressive-like behavior such as the hippocampus.

All in all, it is difficult to pinpoint a single mechanism responsible for the transcriptional changes observed in these regions following PNS. They might be associated with enhanced activation of the hypothalamo–pituitary axis responses (Maccari and Morley-Fletcher, 2007; McGowan and Matthews, 2018; Thayer et al., 2018) as a consequence of the disruption in the negative feedback mechanism, originating from a dysfunctional GR activity or expression. Thereby, changes in the nuclear translocation and transcriptional activity of GRs may promote derangements of hypothalamic–pituitary–adrenal (HPA) axis responsiveness to glucocorticoids, which in turn may contribute to HPA axis hyperactivity (Anacker et al., 2011). Moreover, given the relationships between glucocorticoids and glutamate receptors (Popoli et al., 2011), an overly active HPA-axis activity might drain the pathway of glutamatergic receptor activation, which encourages cortical E/I imbalance at adulthood.

It is conceivable also that the effects of PNS on emotional traits evolve postnatally, with a combination of altered postnatal developmental trajectories of the PFC and AMY and altered offspring’s stress reactivity, which may enhance the propensity to develop an anxiety or depressive disorder later in life.

In summary, the findings of the present study expand our knowledge on the sex differences in the nature and extent of the changes in adult behavior and gene expression of various markers of the E/I balance induced by PNS.

## Data Availability Statement

The raw data supporting the conclusions of this article will be made available by the authors, without undue reservation.

## Ethics Statement

The animal study was reviewed and approved by Committee of Ethics on the Use of Animals (CEUA) – Pontifical Catholic University of Rio Grande do Sul, Brazil (PUCRS).

## Author Contributions

MR, FM, and VB designed the research. FM, KC, VB, AS, LW-S, ST, RO, and ÉK-F performed the research. FM, KC, VB, and AS analyzed the data. MR and RG-O provided the funding and/or supervision, and contributed to the data interpretation and revision of the manuscript. FM wrote the first draft. All authors reviewed and approved the manuscript.

## Conflict of Interest

MR has received compensation as speaker/consultant from the Angelini, Lundbeck, Otzuka, Sumitomo Dainippon Pharma, and Sunovion, and he has received research grants from Sumitomo Dainippon Pharma and Sunovion. The remaining authors declare that the research was conducted in the absence of any commercial or financial relationships that could be construed as a potential conflict of interest.

## Supplementary Material

Supplementary Figure 1 Litter sizes and weights of animals included in the study. **(A)** Summary table depicting litter sizes for each dam of either control or PNS groups and relative sex ratio, along with final number of animals included in the experiment. **(B)** Body weight of control and PNS dams is shown over the course of gestation. **(C)** Comparison between control and PNS litter sizes is graphed. **(D-F)** Body weights of male and female offspring of control and PNS groups, at post-natal day (PND) 1, 14, 21, and 43. Data information: error bars indicate mean ± SEM. For direct comparison of two groups: ^∗∗∗^*p* < 0.001 (two-way ANOVA with Bonferroni’s multiple-comparison test).

Supplementary Figure 2 Analysis of social investigation behaviors in prenatally stressed adult rats. The pre-target session begins with a 3-min habituation session in the arena containing an empty perforated enclosure, followed by a 3-min post-target session in the same arena with the perforated enclosure containing a conspecific rat. **(A)** Total time male rats spent in the perimetral area around the perforated enclosure exploring, touching, or sniffing it. Two-way ANOVA analysis revealed a significant effect of session *F*(1, 93) = 51.24, *p* < 0.0001. **(B**) Total time female rats spent in the perimetral area around the perforated enclosure exploring, touching, or sniffing it. Two-way ANOVA analysis revealed a significant effect of session *F*(1, 70) = 77.93, *p* < 0.0001. **(C)** Total time male rats spent in the opposite corners to the perforated enclosure during both pre- and post-target sessions. Two-way ANOVA analysis revealed a significant effect of stress *F*(1, 94) = 12.05, *p* = 0.0008. **(D)** Total time female rats spent in the opposite corners to the perforated enclosure during both pre- and post-target sessions. Two-way ANOVA analysis revealed a significant effect of stress *F*(1, 70) = 5.982, *p* = 0.0170. **(E)** Data for investigation ratio in male and female rats. Two-way ANOVA analysis revealed a significant effect of sex [*F*(1, 71) = 11.36, *p* = 0.0012]. Data information: symbols represent individual data. ^∗^*p* < 0.05; ^∗∗∗^*p* < 0.001 (two-way ANOVA with Bonferroni’s multiple-comparison test).

Supplementary Figure 3 Analysis of sucrose consumption in prenatally stressed adult rats. **(A)** Total water and sucrose intake. Two-way ANOVA analysis revealed significant effects of stress [*F*(1, 157) = 17.57, *p* < 0.0001] and sex [*F*(3, 157) = 36.05, *p* < 0.0001] for direct comparison of two groups: ^∗∗^*p* < 0.01, ^∗∗∗^*p* < 0.001, ^****^*p* < 0.0.001 Bonferroni’s multiple-comparison test. Data information: symbols represent individual data. Error bars indicate mean ± SEM. (**B**) Total fluid intake during the sucrose preference test. Two-way ANOVA analysis revealed a significant effect of sex [*F*(1, 78) = 44.49, *p* < 0.0001]. For direct comparison of two groups: ^∗∗∗^*p* < 0.001, ^****^*p* < 0.0.001 Bonferroni’s multiple-comparison test. Symbols represent individual data. Error bars indicate mean ± SEM.

Supplementary Figure 4 Analysis of maternal care behavior. **(A)** Number of events of maternal behavior of control and stressed dams. The total of all behavioral categories examined over the first 15 days postnatally is graphed. Data information: symbols represent individual data. Error bars indicate mean ± SEM. Two-way ANOVA revealed no significant effect of type of stress [*F*(1, 111) = 0.003767, *p* = 0.9512]. **(B)** The graph shows the frequency of pups-directed behavior, including dams building the nest, in the nest nursing in any position, licking pups, contact with pups, retrieving the pups or no interaction with pups versus self-directed behavior, involving eating/drinking, self-grooming, off or exit from the nest, from a total of 200 observations per mother (40 observations per mother per day). Data information: symbols represent individual data. Error bars indicate mean ± SEM. Two-way ANOVA revealed a significant effect of type of behavior *F*(9, 180) = 68.68, *p* < 0.0001. For direct comparison of two groups: ^∗∗∗^*p* < 0.001. (Bonferroni’s multiple-comparison test).

Supplementary Figure 5 Analysis of locomotor activity of prenatally stressed adult rats in the open field test. **(A)** Total distance traveled across 5-min period in the arena was measured. Two-way ANOVA analysis revealed significant effects of sex [*F*(1, 78) = 20.40, *p* < 0.0001]. (**B**) Latency to enter the center of the arena was scored. **(C)** The number of entries in the center of the arena is shown. Two-way ANOVA analysis revealed significant effects of sex [*F*(1, 79) = 8.289, *p* = 0.0051]. **(D)** The number of entries in the periphery of the arena is shown. Two-way ANOVA analysis revealed significant effects of sex [*F*(1, 80) = 8.306, *p* = 0.0051] **(E)** Cumulative time spent in the center of the open field across 5-min time is shown. **(F)** Cumulative time spent in the periphery of the open field across 5-min time was measured. Data information: symbols represent individual data. Error bars indicate mean ± SEM. For direct comparison of two groups: ^∗^*p* < 0.05, ^∗∗∗^*p* < 0.001, Bonferroni’s multiple-comparison test.
