# Supplementary figures and images for: Exposure to Prenatal Stress Is Associated With an Excitatory/Inhibitory Imbalance in Rat Prefrontal Cortex and Amygdala and an Increased Risk for Emotional Dysregulation
Source: Front Cell Dev Biol. 2021 Jun 1;9:653384. doi: 10.3389/fcell.2021.653384 (PMC8204112; doi:10.3389/fcell.2021.653384)

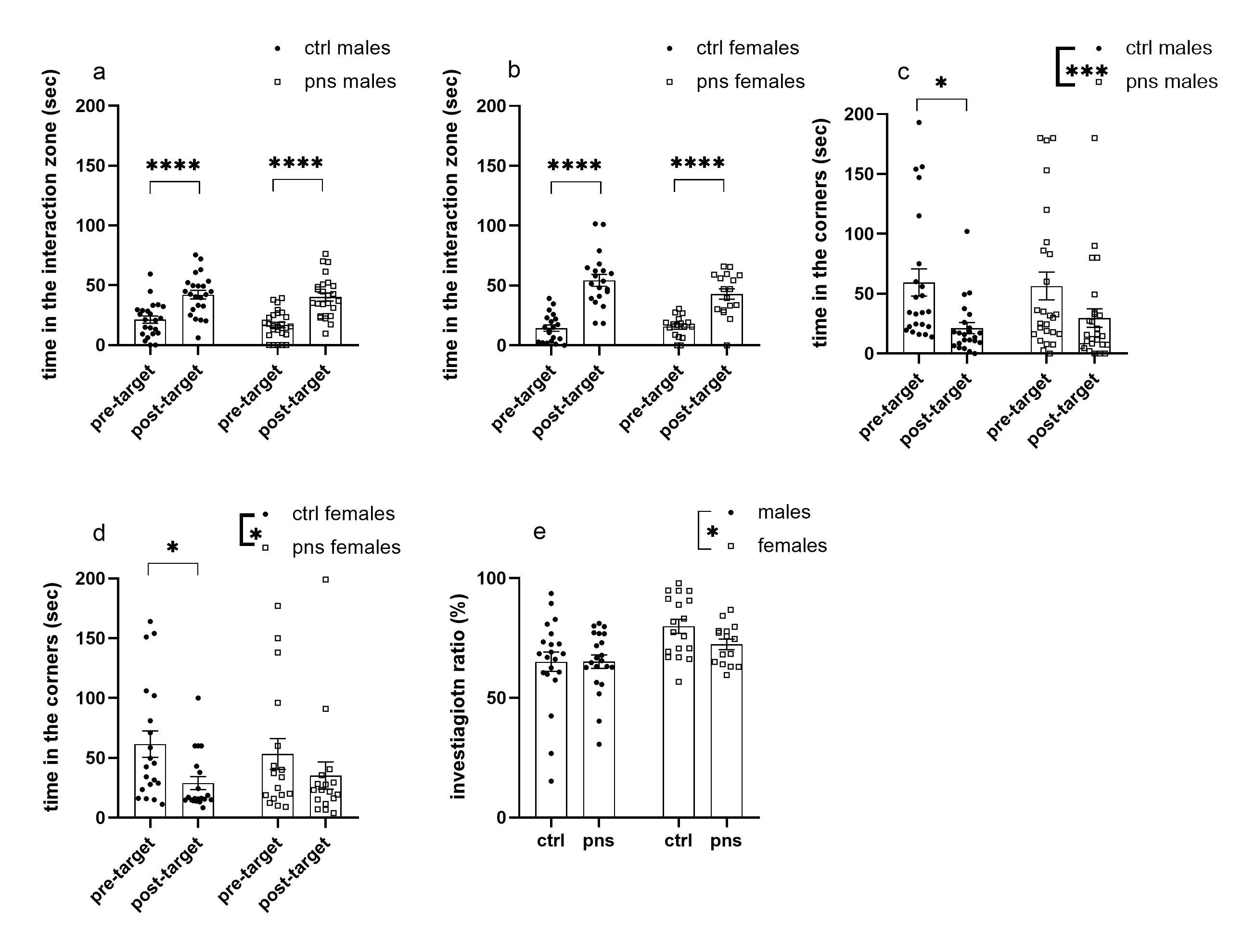

Supplement: Supplementary Figure 1 — Litter sizes and weights of animals included in the study. (A) Summary table depicting litter sizes for each dam of either control or PNS groups and relative sex ratio, along with final number of animals included in the experiment. (B) Body weight of control and PNS dams is shown over the course of gestation. (C) Comparison between control and PNS litter sizes is graphed. (D-F) Body weights of male and female offspring of control and PNS groups, at post-natal day (PND) 1, 14, 21, and 43. Data information: error bars indicate mean ± SEM. For direct comparison of two groups: ∗∗∗p < 0.001 (two-way ANOVA with Bonferroni’s multiple-comparison test). [file Image_1.TIF]

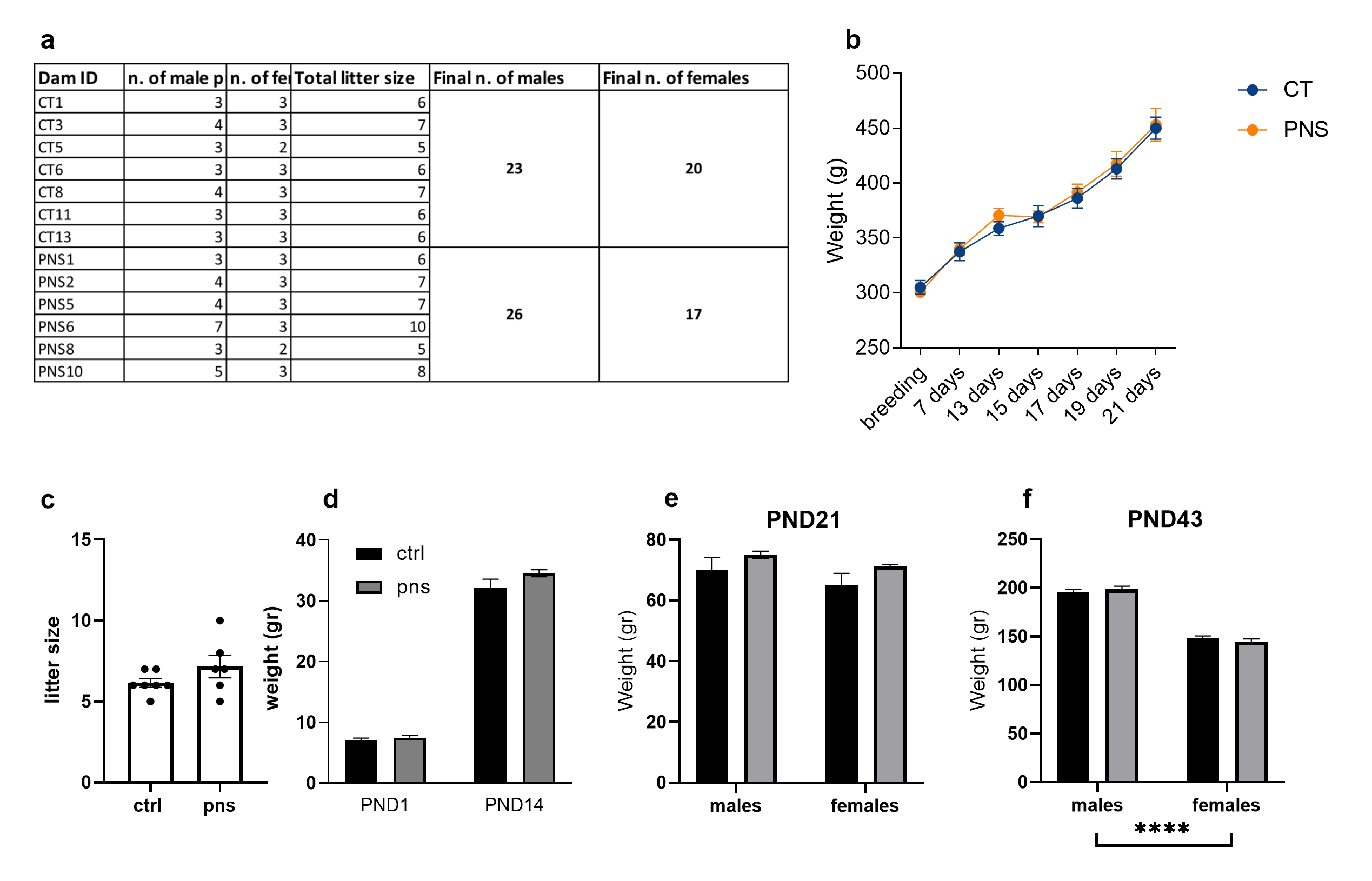

Supplement: Supplementary Figure 2 — Analysis of social investigation behaviors in prenatally stressed adult rats. The pre-target session begins with a 3-min habituation session in the arena containing an empty perforated enclosure, followed by a 3-min post-target session in the same arena with the perforated enclosure containing a conspecific rat. (A) Total time male rats spent in the perimetral area around the perforated enclosure exploring, touching, or sniffing it. Two-way ANOVA analysis revealed a significant effect of session F(1, 93) = 51.24, p < 0.0001. (B) Total time female rats spent in the perimetral area around the perforated enclosure exploring, touching, or sniffing it. Two-way ANOVA analysis revealed a significant effect of session F(1, 70) = 77.93, p < 0.0001. (C) Total time male rats spent in the opposite corners to the perforated enclosure during both pre- and post-target sessions. Two-way ANOVA analysis revealed a significant effect of stress F(1, 94) = 12.05, p = 0.0008. (D) Total time female rats spent in the opposite corners to the perforated enclosure during both pre- and post-target sessions. Two-way ANOVA analysis revealed a significant effect of stress F(1, 70) = 5.982, p = 0.0170. (E) Data for investigation ratio in male and female rats. Two-way ANOVA analysis revealed a significant effect of sex [F(1, 71) = 11.36, p = 0.0012]. Data information: symbols represent individual data. ∗p < 0.05; ∗∗∗p < 0.001 (two-way ANOVA with Bonferroni’s multiple-comparison test). [file Image_2.TIF]

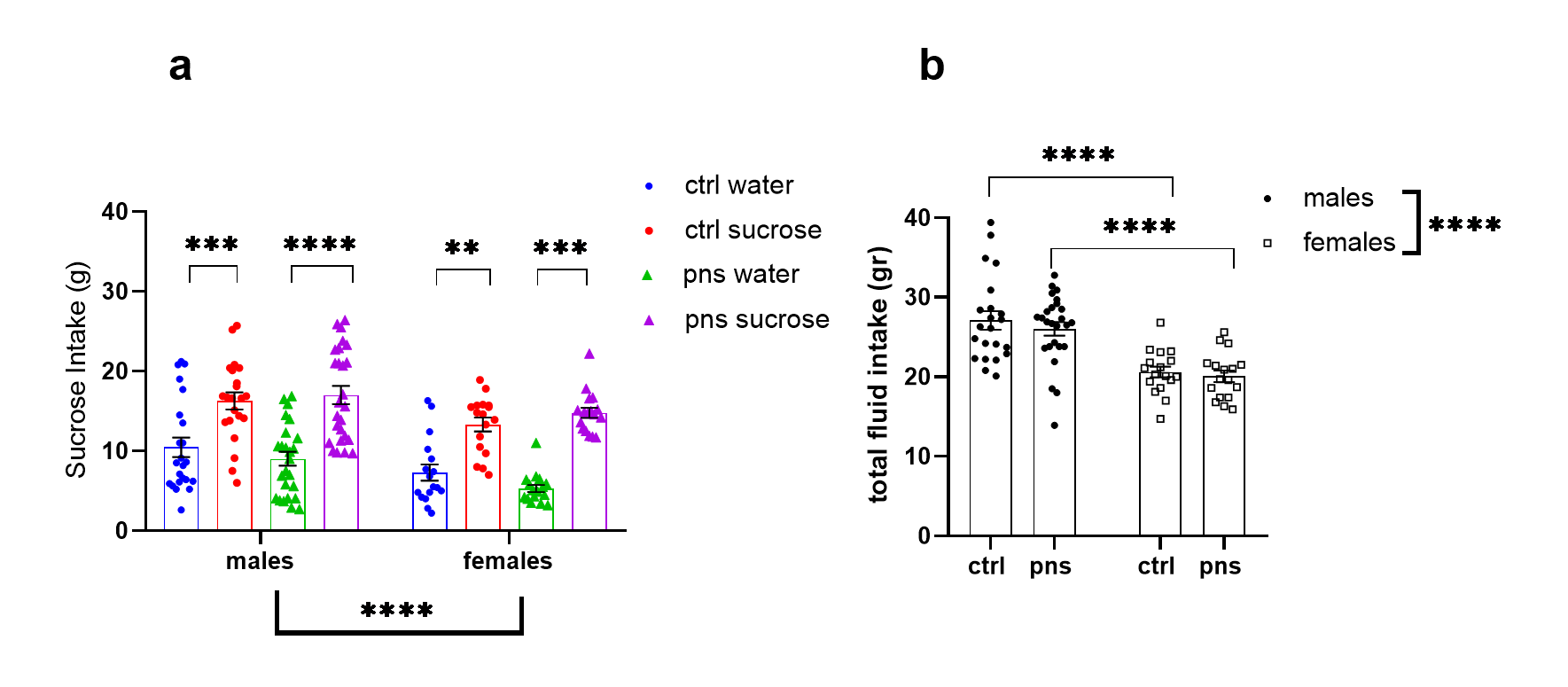

Supplement: Supplementary Figure 3 — Analysis of sucrose consumption in prenatally stressed adult rats. (A) Total water and sucrose intake. Two-way ANOVA analysis revealed significant effects of stress [F(1, 157) = 17.57, p < 0.0001] and sex [F(3, 157) = 36.05, p < 0.0001] for direct comparison of two groups: ∗∗p < 0.01, ∗∗∗p < 0.001, ****p < 0.0.001 Bonferroni’s multiple-comparison test. Data information: symbols represent individual data. Error bars indicate mean ± SEM. (B) Total fluid intake during the sucrose preference test. Two-way ANOVA analysis revealed a significant effect of sex [F(1, 78) = 44.49, p < 0.0001]. For direct comparison of two groups: ∗∗∗p < 0.001, ****p < 0.0.001 Bonferroni’s multiple-comparison test. Symbols represent individual data. Error bars indicate mean ± SEM. [file Image_3.TIF]

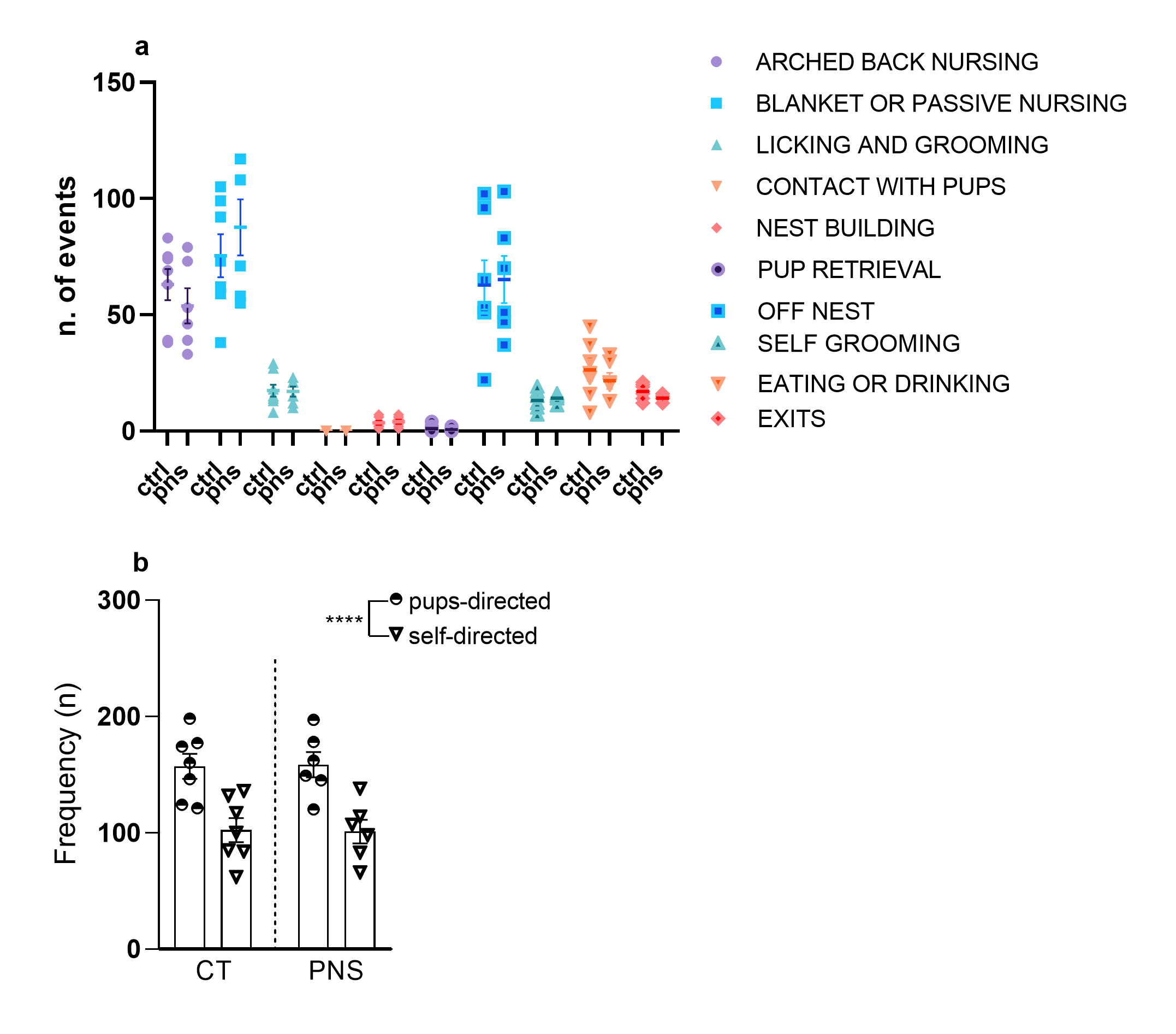

Supplement: Supplementary Figure 4 — Analysis of maternal care behavior. (A) Number of events of maternal behavior of control and stressed dams. The total of all behavioral categories examined over the first 15 days postnatally is graphed. Data information: symbols represent individual data. Error bars indicate mean ± SEM. Two-way ANOVA revealed no significant effect of type of stress [F(1, 111) = 0.003767, p = 0.9512]. (B) The graph shows the frequency of pups-directed behavior, including dams building the nest, in the nest nursing in any position, licking pups, contact with pups, retrieving the pups or no interaction with pups versus self-directed behavior, involving eating/drinking, self-grooming, off or exit from the nest, from a total of 200 observations per mother (40 observations per mother per day). Data information: symbols represent individual data. Error bars indicate mean ± SEM. Two-way ANOVA revealed a significant effect of type of behavior F(9, 180) = 68.68, p < 0.0001. For direct comparison of two groups: ∗∗∗p < 0.001. (Bonferroni’s multiple-comparison test). [file Image_4.TIF]

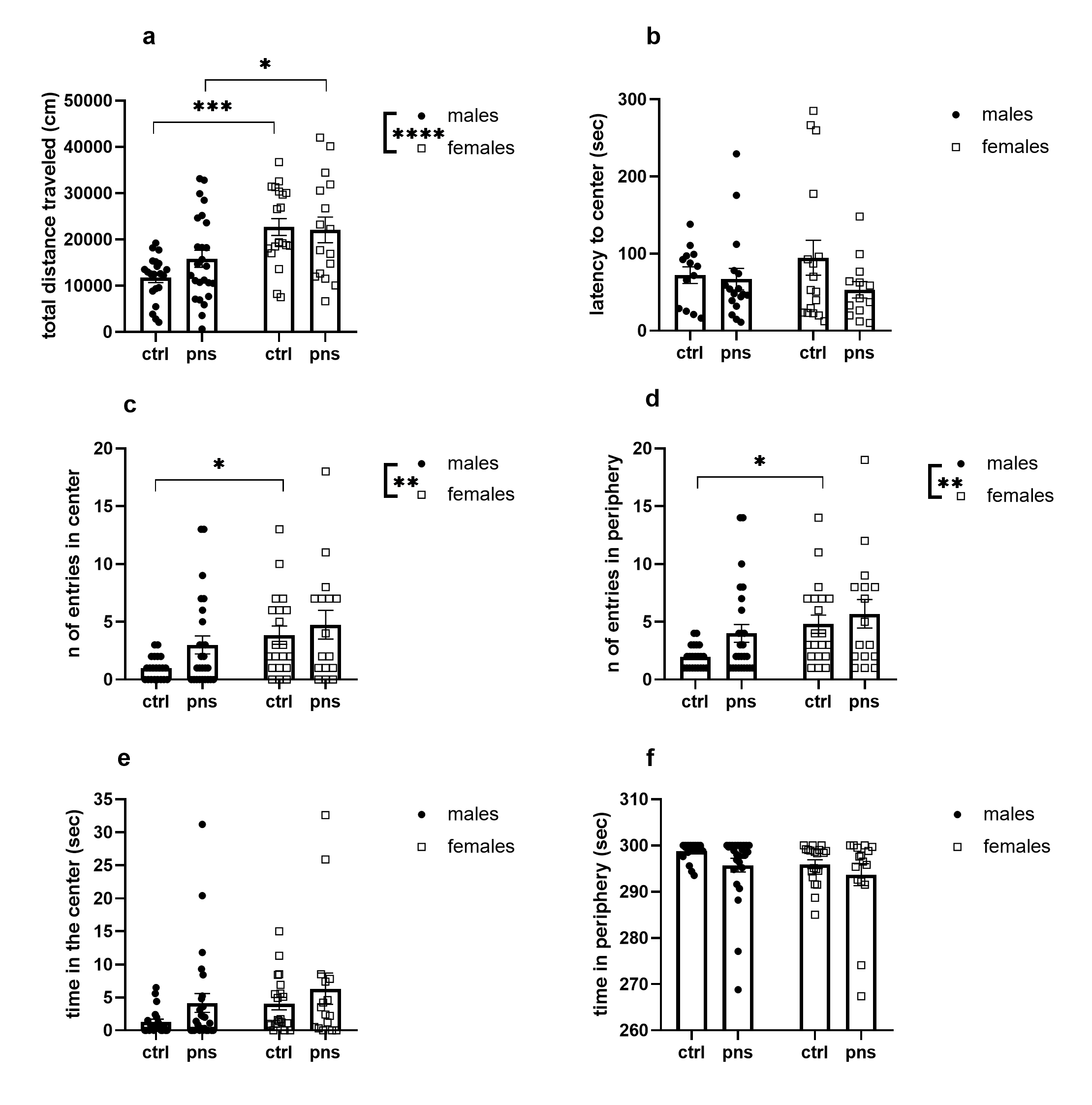

Supplement: Supplementary Figure 5 — Analysis of locomotor activity of prenatally stressed adult rats in the open field test. (A) Total distance traveled across 5-min period in the arena was measured. Two-way ANOVA analysis revealed significant effects of sex [F(1, 78) = 20.40, p < 0.0001]. (B) Latency to enter the center of the arena was scored. (C) The number of entries in the center of the arena is shown. Two-way ANOVA analysis revealed significant effects of sex [F(1, 79) = 8.289, p = 0.0051]. (D) The number of entries in the periphery of the arena is shown. Two-way ANOVA analysis revealed significant effects of sex [F(1, 80) = 8.306, p = 0.0051] (E) Cumulative time spent in the center of the open field across 5-min time is shown. (F) Cumulative time spent in the periphery of the open field across 5-min time was measured. Data information: symbols represent individual data. Error bars indicate mean ± SEM. For direct comparison of two groups: ∗p < 0.05, ∗∗∗p < 0.001, Bonferroni’s multiple-comparison test. [file Image_5.TIF]
