# Supplementary material for: Exposure to Prenatal Stress Is Associated With an Excitatory/Inhibitory Imbalance in Rat Prefrontal Cortex and Amygdala and an Increased Risk for Emotional Dysregulation
Source: Front Cell Dev Biol. 2021 Jun 1;9:653384. doi: 10.3389/fcell.2021.653384 (PMC8204112; doi:10.3389/fcell.2021.653384)
Supplement: Supplementary file 6 [file Data_Sheet_1.PDF]

Alpha	Area	Gene	stress		sex		interaction		Bonferroni's multiple comparisons test			
			**F (DFn, DFd)**	**P value**	**F (DFn, DFd)**	**P value**	**F (DFn, DFd)**	**P value**	**control vs. PNS males P value**	**control vs. PNS females P value**	**males vs. females control P value**	**males vs. females PNS P value**
0,05	PFC	*Grin1*	F (1, 48) = 1.821	P=0.1835	F (1, 48) = 2.686	P=0.1078	F (1, 48) = 3.397	P=0.0715	>0.9999	0,082	0,0439	>0.9999
		Grin2a	F (1, 76) = 3.859	P=0.0531	F (1, 76) = 0.7988	P=0.3743	F (1, 76) = 1.306	P=0.2568	0,0384	>0.9999	>0.9999	0,3352
		Grin2b	F (1, 74) = 5.859	P=0.0180	F (1, 74) = 4.507	P=0.0371	F (1, 74) = 3.789	P=0.0554	0,0029	>0.9999	0,0099	>0.9999
		Grin2a/Grin2b ratio	F (1, 68) = 19.72	P<0.0001	F (1, 68) = 6.732	P=0.0116	F (1, 68) = 3.313	P=0.0731	<0.0001	0,1668	0,004	>0.9999
		vGlut1	F (1, 56) = 1.155	P=0.2872	F (1, 56) = 3.628	P=0.0620	F (1, 56) = 10.84	P=0.0017	0,008	0,2233	0,7393	0,0003
		vGat	F (1, 74) = 3.831	P=0.0541	F (1, 74) = 1.917	P=0.1703	F (1, 74) = 0.0660	P=0.7979	0,4003	0,2918	0,4667	0,8852
		vGlut1/vgat ratio	F (1, 53) = 0.1979	P=0.6582	F (1, 53) = 0.6097	P=0.4384	F (1, 53) = 8.116	P=0.0062	0,0555	0,1708	0,3634	0,0137
		Gad1/67	F (1, 74) = 0.01104	P=0.9166	F (1, 74) = 0.9131	P=0.3424	F (1, 74) = 3.925	P=0.0513	0,3052	0,349	0,9217	0,0913
		Arc/Arg 3.1	F (1, 68) = 3.505	P=0.0655	F (1, 68) = 1.816	P=0.1823	F (1, 68) = 1.710	P=0.1954	0,0316	>0.9999	0,1092	>0.9999
		Npas4	F (1, 66) = 7.612	P=0.0075	F (1, 66) = 1.691	P=0.1980	F (1, 66) = 4.115	P=0.0465	0,001	>0.9999	0,0339	>0.9999
		Zif268	F (1, 68) = 0.9356	P=0.3369	F (1, 68) = 6.724	P=0.0116	F (1, 68) = 2.051	P=0.1567	>0.9999	0,2294	0,7882	0,0159
	AMY	*Grin1*	F (1, 59) = 4.360	P=0.0411	F (1, 59) = 7.739	P=0.0072	F (1, 59) = 1.068	P=0.3055	0,0609	0,9218	0,0226	0,4126
		vGlut1	F (1, 59) = 4.929	P=0.0303	F (1, 59) = 0.4813	P=0.4905	F (1, 59) = 1.603	P=0.2104	0,0271	>0.9999	0,3867	>0.9999
		vGat	F (1, 52) = 5.122	P=0.0278	F (1, 52) = 1.136	P=0.2913	F (1, 52) = 0.6890	P=0.4103	0,0661	0,6324	>0.9999	0,3443
		vGlut1/vgat ratio	F (1, 51) = 3.120	P=0.0833	F (1, 51) = 6.639e-0[illegible]	P=0.9935	F (1, 51) = 0.7325	P=0.3961	0,1347	>0.9999	>0.9999	>0.9999
		Gad1/67	F (1, 52) = 15.94	P=0.0002	F (1, 52) = 2.616	P=0.1118	F (1, 52) = 1.895	P=0.1745	0,0005	0,1572	>0.9999	0,0758
		Arc/Arg 3.1	F (1, 46) = 4.090	P=0.0490	F (1, 46) = 14.50	P=0.0004	F (1, 46) = 5.244	P=0.0267	0,0093	>0.9999	0,0003	0,531
		Npas4	F (1, 48) = 0.3022	P=0.5851	F (1, 48) = 5.894	P=0.0190	F (1, 48) = 0.2422	P=0.6249	0,9588	>0.9999	0,1168	0,2959
		Zif268	F (1, 50) = 8.234	P=0.0060	F (1, 50) = 2.677	P=0.1081	F (1, 50) = 3.842	P=0.0556	>0.9999	0,0165	>0.9999	0,0022

Supplementary table 1: Effect of the stress, sex and stress X sex interaction in the Two-way ANOVA analysis
